# PyTEA-O: a Python implementation of Two-Entropies Analysis for protein sequence variation analysis

**DOI:** 10.1093/bioinformatics/btag043

**Published:** 2026-02-04

**Authors:** Rosan C M Kuin, Alexander T Julian, Jagriti Chander, Sunah Lee, Gerard J P van Westen

**Affiliations:** Computational Drug Discovery, Division of Medicinal Chemistry, Leiden Academic Centre of Drug Research, Leiden University, 2333 CC Leiden, The Netherlands; Department of Biology, Illinois Institute of Technology, Chicago, IL 60616, United States; Department of Biology, Illinois Institute of Technology, Chicago, IL 60616, United States; Department of Biology, Illinois Institute of Technology, Chicago, IL 60616, United States; Computational Drug Discovery, Division of Medicinal Chemistry, Leiden Academic Centre of Drug Research, Leiden University, 2333 CC Leiden, The Netherlands

## Abstract

**Motivation:**

Protein sequence variation analysis is a topic of broad interest in drug discovery and protein engineering to support modulation of protein function for diverse biotechnological and therapeutic applications. To assist in the analysis of multiple sequence alignments (MSAs) and identify residues that account for protein function specificity, computational tools have been developed. Yet, existing programs often omit consideration of amino acid properties, flexibility beyond fixed webserver interfaces, accessible source code, or compatibility with small MSAs.

**Results:**

To address these limitations, we present PyTEA-O, a Python implementation of Two-Entropies Analysis that has been developed to be easy to use for the analysis of protein sequence variation. To help users analyze the MSA and screen for residues of interest, we generate modifiable and intuitive visualizations. These visualizations, together with a scoring approach for identifying alignment positions with (dis-)similar physicochemical properties, presents a powerful tool for sequence variability analysis. To demonstrate its capabilities, we present a case study based on the deubiquitinase OTUD7B (Cezanne) where we identify a crucial position that modulates its affinity for its substrate.

**Availability and implementation:**

PyTEA-O is available at https://github.com/CDDLeiden/PyTEA-O/ and archived via Zenodo (https://doi.org/10.5281/zenodo.15914598).

## 1 Introduction

Proteins are essential to all biological systems, serving as the key drivers of various biological processes (Agarwal 2006, Westermarck *et al*. 2013, Morris, Black and Stollar 2022). The amino acid sequence of a protein dictates their specific three-dimensional structure, resulting in a wide range of functions, with this class of macromolecules being involved in virtually every cellular process (Lodish and Berk 2021). The biological role of proteins are dependent on their three-dimensional conformation such that their functionality can be inferred by structural similarity, even in cases with significant sequence divergence (Julian *et al*. 2021). Because alterations in an amino acid sequence can alter protein function, protein sequence variation analysis is a broad topic in drug development (Fram *et al.* 2024). Examples include modulating protein function for various biotechnological and therapeutic applications (Jäckel *et al.* 2010), as well as identifying positions associated with vaccine resistance (Walter *et al*. 2021).

Due to the costly and labor-intensive process of experimentally determining such function defining residues, various computational tools based on multiple sequence alignments (MSAs) have been developed to prioritize variants and guide experimental design (de Melo-Minardi *et al*. 2010, Chagoyen *et al*. 2016).

Previously, a Two-Entropies Analysis (TEA) was developed to identify specificity-determining residues in protein families using Shannon Entropy to measure sequence conservation, based on a MSA (Ye *et al.* 2006, Ye *et al*. 2008). Initially users needed to manually specify familial divisions, resulting in potentially non-reproducible results. This shortcoming was later resolved by modifying the algorithm to average the Shannon Entropy at each position for an objective (user-bias free) division across all protein families. The implementation, now called TEA-O, automatically divides the provided sequences into subgroups, and calculates the sequence conservation for each subgroup, based on the Shannon Entropy. TEA and TEA-O have been successfully applied in several studies to study protein sequence variation, including an analysis of somatic mutations in GPCRs (Bongers *et al.* 2022) and identification of fidelity determining residues in bacterial DNA polymerases (Kuin *et al*. 2025).

Here, we used Python to re-implement the original TEA-O algorithm in a more versatile code in a tool aptly named PyTEA-O. These enhancements incorporate a new sub-group definition based on taxonomy, a scoring scheme based on physicochemical properties, a local implementation of the Unweighted Pair Group Method with Arithmetic Mean (UPGMA) clustering algorithm to reduce variations between differing clustering tools, and the creation of detailed publication-ready visualizations. PyTEA-O is fast, open-sourced, easy to use for scientists of all backgrounds, and designed to be easily extendable, allowing the user to implement other scores or ranking methods of their choice.

## 2 Material and methods

The original algorithm with full details are described in the original publication (Ye *et al*. 2008). We expand on this implementation through the addition of several new features that broaden its applicability, which we describe here.

### 2.1 Software and requirements

PyTEA-O is an open-source Python package (version 3.10). Starting from an MSA, the phylogenetic tree and Shannon Entropy calculations are automatically generated, with no additional user input needed. With a minimum input of just two sequences, it remains suitable for projects with limited data.

### 2.2 Taxonomic annotation from UniProt identifiers

In some cases, restricting sequences to specific taxonomic groups is necessary. For example, when studying a protein family unique to certain bacteria, excluding similar sequences from other domains of life may be essential. Additionally, over-representation of certain taxa in a MSA may introduce bias, potentially skewing variant analysis. To address this, the full taxonomic lineage for each sequence can be retrieved from NCBI (Sayers *et al.* 2025). This is done by downloading the taxonomy and accession mapping files from NCBI, then mapping the MSA sequence accessions to their corresponding taxonomic IDs.

### 2.3 Scoring scheme based on physicochemical properties

**Figure 1:**
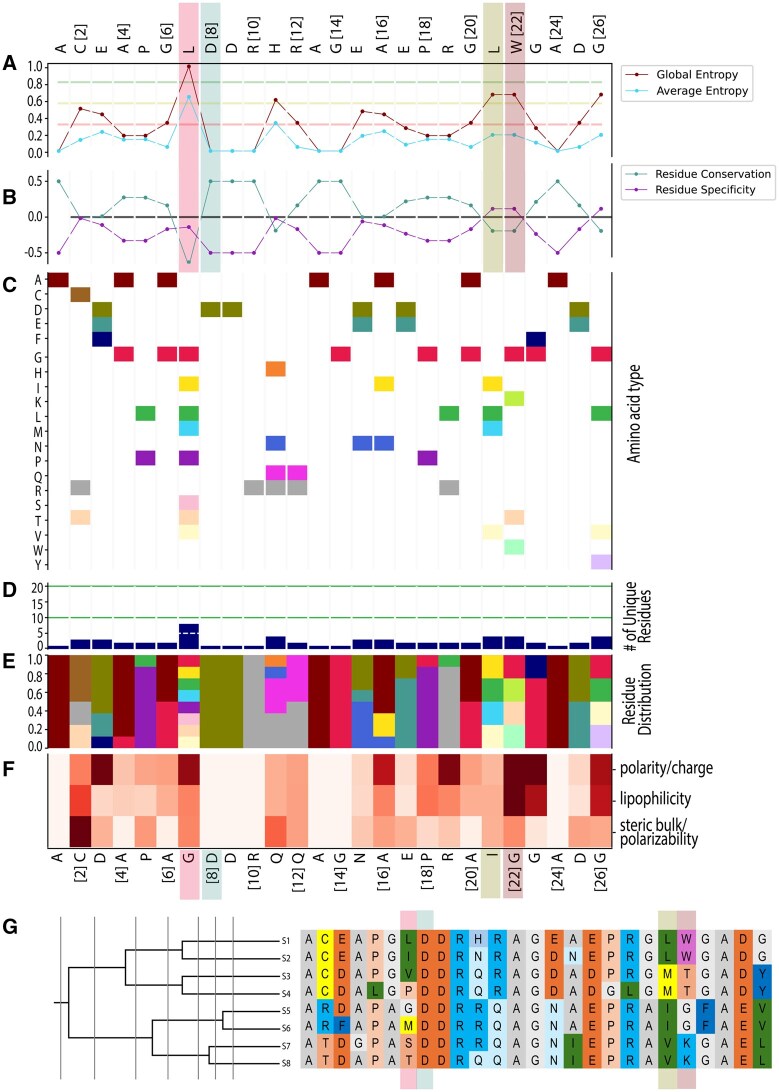
Visualization of entropy calculations and residue scoring per residue in TEA-O mode. The sequence displayed above subfigure A represents the reference sequence (S1), while the sequence below subfigure F shows the most conserved residues at each alignment position. Highlighted alignment positions include: position 7 (non-conserved), position 8 (fully conserved), and positions 21 and 22 (specificity-determining). Among these, position 21 has similar physicochemical properties across sequences, while position 22 shows greater divergence. (A) Shannon Entropy values. Global and average Shannon Entropy is indicated by the maroon and blue line, respectively. (B) Specificity/conservation scoring. Residue conservation (maintenance of a residue across all sequences) and specificity (maintenance of a residue within a subfamily, but not across all sequences) are shown in blue and pink, respectively. (C) Residue presence matrix. Each amino acid is represented by a different color, while white indicates its absence in the MSA. (D) Residue variants. Total number of different amino acids present among the 20 natural amino acids. (E) Residue frequency distribution. Following the color scheme of (C), displays the frequency that each residue appears at a given position within the MSA. (F) Z-scale heatmap. Shows the standard deviations for Z-scale 1–3, representing lipophilicity, steric bulk/polarizability, and polarity/charge, respectively. Darker colored squares indicate greater variability in residue properties at a given position. (G) The phylogenetic tree illustrates the different subgroup definitions used for the TEA. Vertical grey lines indicate the divisions produced by the UPGMA algorithm.

Since information entropy treats amino acids as independent symbols, it overlooks their unique characteristics, such as size and functional groups. To address this, we implemented a scoring metric that takes amino acid physiochemical properties into account using descriptor variables that are based on experimental data, named Z-scales (Sandberg *et al.* 1998). These descriptors are derived from a principal component analysis applied to a large set of experimental data. Each Z-scale is a principal component of the analysis and addresses lipophilicity, steric bulk/polarizability, and polarity/charge (Z-scale 1–3, respectively) (Sandberg *et al.* 1998). Despite having a fourth and fifth Z-scale that relates to electronegativity, heat of formation, electrophilicity and hardness, these values are less intuitive to interpret (van Westen *et al.* 2013), and moreover, the variance described by these scales is low (13% and 6%, respectively) (Sandberg *et al.* 1998). As such, these scales are not included in PyTEA-O. To obtain the Z-scale values, the standard deviation of each Z-scale is calculated for each position in the MSA, as such, positions with greater diversity in these descriptors will show higher variability (Fig. 1, available as supplementary data at *Bioinformatics* online) Additionally, users can supply a custom scoring scheme based on other amino acid properties or domain-specific metrics to allow for more flexibility depending on the research question.

### 2.4 Visualization of sequence variation and residue properties

To better assist the analysis of individual positions of the MSA, we included additional visualizations, complementing those from the original publication (Ye *et al*. 2008). Using only the MSA as input, a figure detailing multiple residue-level metrics, including entropy scores, conservation/specificity assessments, residue presence, amino acid diversity, residue distributions, and physicochemical variability based on Z-scale properties, is produced (Fig. 1). These visualizations provide users with an intuitive tool to interpret sequence variation, identify potential alignment issues and guide further hypothesis generation.

### 2.5 Hardware

Entropy calculations were run on a server running Rocky Linux 8 (Green Obsidian), equipped with two Intel® Xeon® E5-2650 v4 CPUs @ 2.20 GHz (2x12 cores) and 512 GB RAM. All runs used 12 threads.

## 3 Results

### 3.1 Runtime analysis

To evaluate the tool’s performance across MSAs of differing sizes, we generated alignments with varying lengths and numbers of sequences. Results for the UPGMA and TEA calculations are summarized in Tables 1 and 2, available as supplementary data at *Bioinformatics* online. Our results demonstrate that for a substantially large MSA (5000 sequences and 5000 residues in length), the full pipeline, from subgroup creation to output visualization, completes in under 35 min.

### 3.2 Validation of PyTEA-O on synthetic dataset

The first step in confirming the correct implementation of the PyTEA-O was to reproduce prior results. We applied our algorithm to the original synthetic dataset and recreated the results (Fig. 2, available as supplementary data at *Bioinformatics* online) (Ye *et al.* 2008). Overall, our results are nearly identical to those reported in the original publication, with only minor differences that are likely due to small variations in class assignments and these do not hamper the functionality or interpretability of the implementation.

### 3.3 Using PyTEA-O to identify rheostat positions in OTUD7B

OTUD7B, also known as Cezanne, is a member of the ovarian tumor domain-containing deubiquitinase family. OTUD7B plays a critical role in cell signaling pathways, particularly through its specific cleavage of Lys11-linked polyubiquitin chains (Mevissen *et al.* 2016). Structural studies have revealed a complex and flexible mechanism behind OTUD7B’s catalytic specificity. In particular, regions near the active site change shape during the enzymatic cycle (Mevissen *et al.* 2016). In addition, OTUD7B’s catalytic activity can be impacted by individual amino acid changes, known as rheostat positions. At these sites, different substitutions result in a gradation of functional outcomes, rather than an all-or-nothing effect (Fenton *et al.* 2022). For instance, this concept has been studied in human aldolase A, where substitutions at a distal position (I98) shifted both cooperativity and substrate affinity, while leaving the overall structure mostly intact (Fenton *et al.* 2022). These outcomes suggest that rheostat positions modulate protein conformational ensembles and/or dynamics rather than structural integrity per se. Identifying such positions in regulatory enzymes like OTUD7B could reveal evolutionary mechanisms for fine-tuning function and provide useful targets for therapeutic or engineering applications.

To identify rheostat positions, we retrieved the wild-type human OTUD7B sequence (UniProt ID: Q6GQQ9) (UniProt Consortium 2025) and obtained 5000 homologous sequences from NCBI BLAST, excluding synthetic constructs (Altschul *et al.* 1990). Sequences were aligned using MAFFT (v7.490) (Katoh *et al.* 2002) using parameters optimized for large datasets.

Candidate rheostat positions were selected based on high entropy and presence of at least 10 distinct amino acid substitutions across the position. We avoided terminal positions and known catalytic or substrate-binding residues (Table 3, available as supplementary data at *Bioinformatics* online). Among the identified residues, we selected position 657 due to its proximity to a functionally important structural region.

Eleven naturally occurring amino acid substitutions were identified at position 657: I (wild-type), A, E, G, L, M, N, P, S, T, and V. Each mutant sequence was modeled using the HDOCK webserver (http://hdock.phys.hust.edu.cn/) (Huang and Zou 2008, 2014, Yan *et al.* 2017, 2020), with OTUD7B as the receptor and Lys11-linked diubiquitin (PDB ID: 5GOC) (Gao *et al.* 2016) as the ligand. We recorded docking and confidence scores, and ligand Root Mean Squared Distances (RMSD). The model of the enzyme-substrate interaction was analyzed using the PRODIGY webserver (https://rascar.science.uu.nl/prodigy/) (Vangone and Bonvin 2015, Xue *et al.* 2016) to predict thermodynamic binding parameters: Δ*G* and *K*_d_.

The complete output of PyTEA-O is shown in Fig. 3, available as supplementary data at *Bioinformatics* online and the structural modeling results of position 657 are shown in Table 4, available as supplementary data at *Bioinformatics* online. We note that the wild-type I657 yielded a docking score of −234.89. Multiple substitutions: A(−245.11), L(−259.41), M(−238.60), N(−272.40), and P(−269.81) produced more negative docking scores, indicating a more favorable binding mode. In addition, all five substitutions show confidence scores above 0.7, indicating that the molecules are very likely to bind (Huang and Zou 2008, 2014). As controls, we modeled a catalytic-site mutant (D191L) that shows the least favourable binding free energy Δ*G*, while E448 a distant substitution with no expected functional change shows no effect.

PyTEA-O proved highly effective in narrowing down candidate rheostat positions. Position 657 in OTUD7B, selected based on evolutionary signals from PyTEA-O, was validated *in silico* through structural docking and binding energy estimation to assess its functional impact. While HDOCK does not model catalysis or capture conformation ensemble shifts, its docking score provides a useful proxy for changes in substrate-binding potential. The variation in predicted affinities across natural substitutions supports the idea that position 657 modulates binding efficiency, consistent with rheostat behavior. These predictions were complemented by binding free energy (Δ*G*) and dissociation constant (K_d_) predictions from PRODIGY, which enabled thermodynamic quantification of binding affinity. The Δ*G* values ranged from −7.9 to −11.7 kcal/mol and the corresponding *K*_d_ values from 10^−9^ M to 10^−5^ M, reflecting a wide spectrum of affinity changes (Table 4, available as supplementary data at *Bioinformatics* online). Substitutions L, A, M, and N, enhanced binding significantly relative to the wild-type, while P shows reduced affinity. One plausible explanation is that L, A, M, and N introduce side chains that are hydrophobic or conformationally compatible with the local environment, thereby stabilizing the substrate-binding interface. In contrast, P is known as a structural disruptor: its cyclic side chain can introduce rigidity and distort local secondary structure, which may interfere with optimal positioning of residues at the binding site and thus reduce affinity. The graded distribution of binding strength across substitutions aligned with the functional definition of rheostat position, one that tunes, rather than switches, activity. In contrast to traditional mutational analyses requiring extensive site-directed mutagenesis and biochemical assays, this pipeline, using PyTEA-O as initial filtering step, offers a streamlined approach offering time and resource efficiency.

## 4 Conclusion

Shannon Entropy has been widely used for exploring protein sequence variation (Oliveira *et al.* 2003, Bywater 2015). Building on this principle, a Two-Entropies Analysis was presented (Ye *et al.* 2006; Ye *et al*. 2008). Here, we describe PyTEA-O, a fast, open-source, and user-friendly Python implementation of Two-Entropies Analysis. PyTEA-O can be used for exploring protein sequence variation and identification of functional residues. Through a case study, we demonstrate its practical utility in identifying rheostat positions and interpreting sequence conservation. While the accuracy of results depends on the quality and diversity of the input MSA, PyTEA-O offers detailed visualizations to assist users in interpretating results. Ultimately, this tool provides a flexible framework for uncovering sequence-function patterns and guiding hypothesis development with minimal user-input.

## Supplementary Material

btag043_Supplementary_Data

## Data Availability

The source code is available at https://github.com/CDDLeiden/PyTEA-O/. All code and data are archived via Zenodo (https://doi.org/10.5281/zenodo.15914598).
