# Supplementary material for: PyTEA-O: a Python implementation of Two-Entropies Analysis for protein sequence variation analysis
Source: Bioinformatics. 2026 Feb 4;42(2):btag043. doi: 10.1093/bioinformatics/btag043 (PMC12910380; doi:10.1093/bioinformatics/btag043)
Supplement: btag043_Supplementary_Data [file btag043_supplementary_data.docx]

# *Supplementary Information*

**PyTEA-O: a Python implementation of Two-Entropies Analysis for protein sequence variation analysis**

R.C.M. Kuin^1†^, A.T. Julian^2†^, J. Chander^2^, S. Lee^2^, G.J.P. van Westen^1*^

**Supplementary Figure 1**. Example positions of a MSA for illustrating Z-scale calculations. **(A)** Residues are colored based on their conservation, with positions 3 and 4 being specificity-determining residues. **(B)** Residues are colored according to molecular weight, using a gradient from blue (low molecular weight) to red (high molecular weight). **(C)** Heatmap showing Z-scale calculations for position 1-4. Positions 3 and 4 are both specificity-determining residues, with an overall Shannon Entropy and average Shannon Entropy of 0. However, position 4 shows greater variability in residue size. This change in bulk is captured in Z-scale 2 (steric bulk/polarizability) and is visually apparent in the heatmap as increased variation in color intensity at position 4, demonstrating how Z-scales can be used to filter residues based on different physicochemical properties.
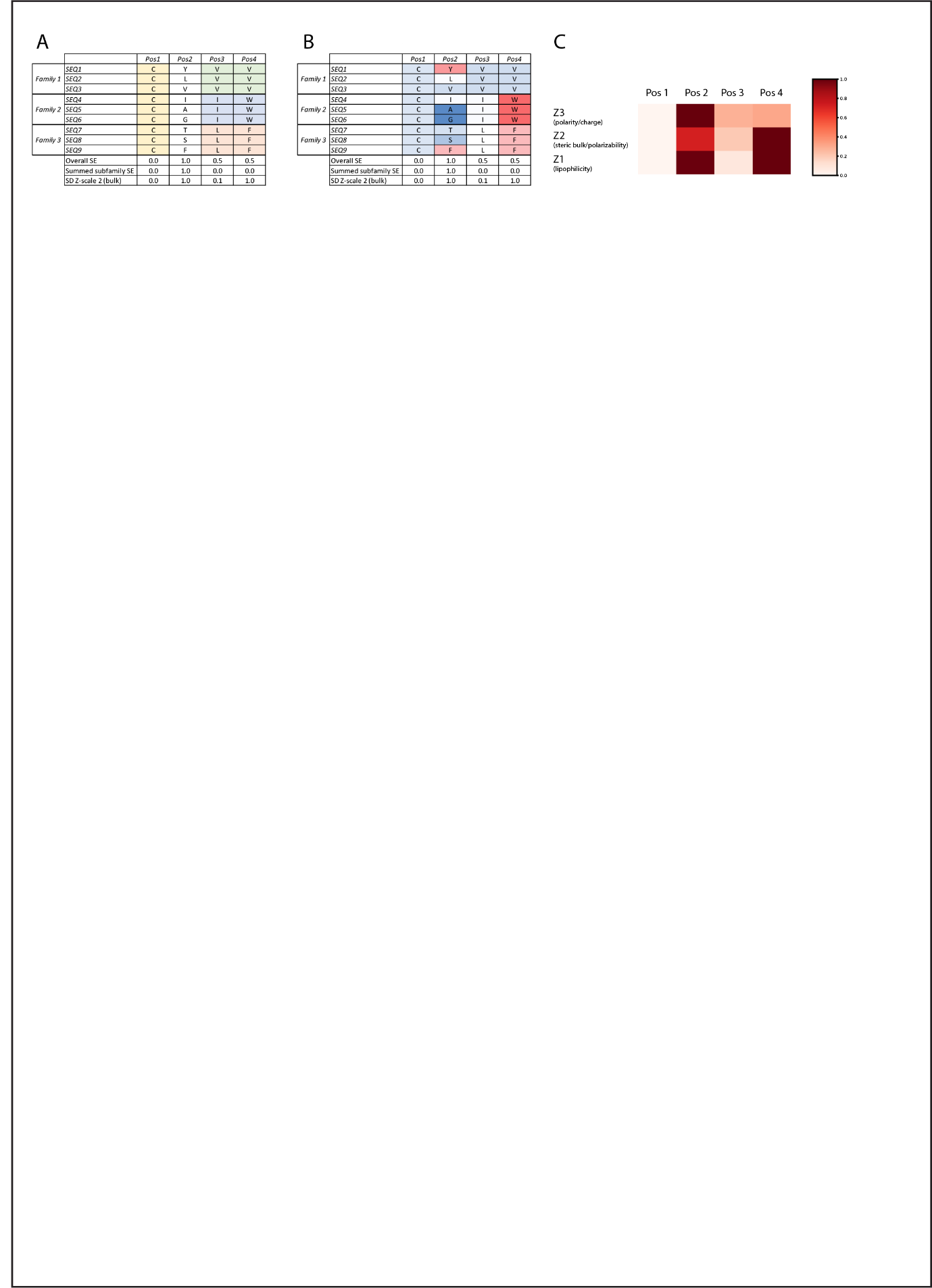


**Supplementary Table 1**. Runtime (in seconds) for UPGMA tree construction across multiple MSA sizes. Rows indicate the number of sequences; columns indicate the sequence length.

|  | **Length of each sequence** | | | |
| --- | --- | --- | --- | --- |
| **Number of Sequences** | **500** | **1000** | **2000** | **5000** |
| **500** | 5 | 6 | 6 | 7 |
| **1000** | 21 | 22 | 22 | 24 |
| **2000** | 84 | 87 | 87 | 93 |
| **5000** | 537 | 545 | 546 | 567 |

**Supplementary Table 2**. Runtime (in seconds) for TEA calculations across multiple MSA sizes. Rows indicate the number of sequences; columns indicate the sequence length. Timings reflect the total runtime from the initiation of TEA calculations through to the generation of output visualizations.

|  | **Length of each sequence** | | | |
| --- | --- | --- | --- | --- |
| **Number of Sequences** | **500** | **1000** | **2000** | **5000** |
| **500** | 57 | 104 | 230 | 531 |
| **1000** | 70 | 128 | 258 | 645 |
| **2000** | 97 | 175 | 340 | 835 |
| **5000** | 192 | 336 | 597 | 1434 |


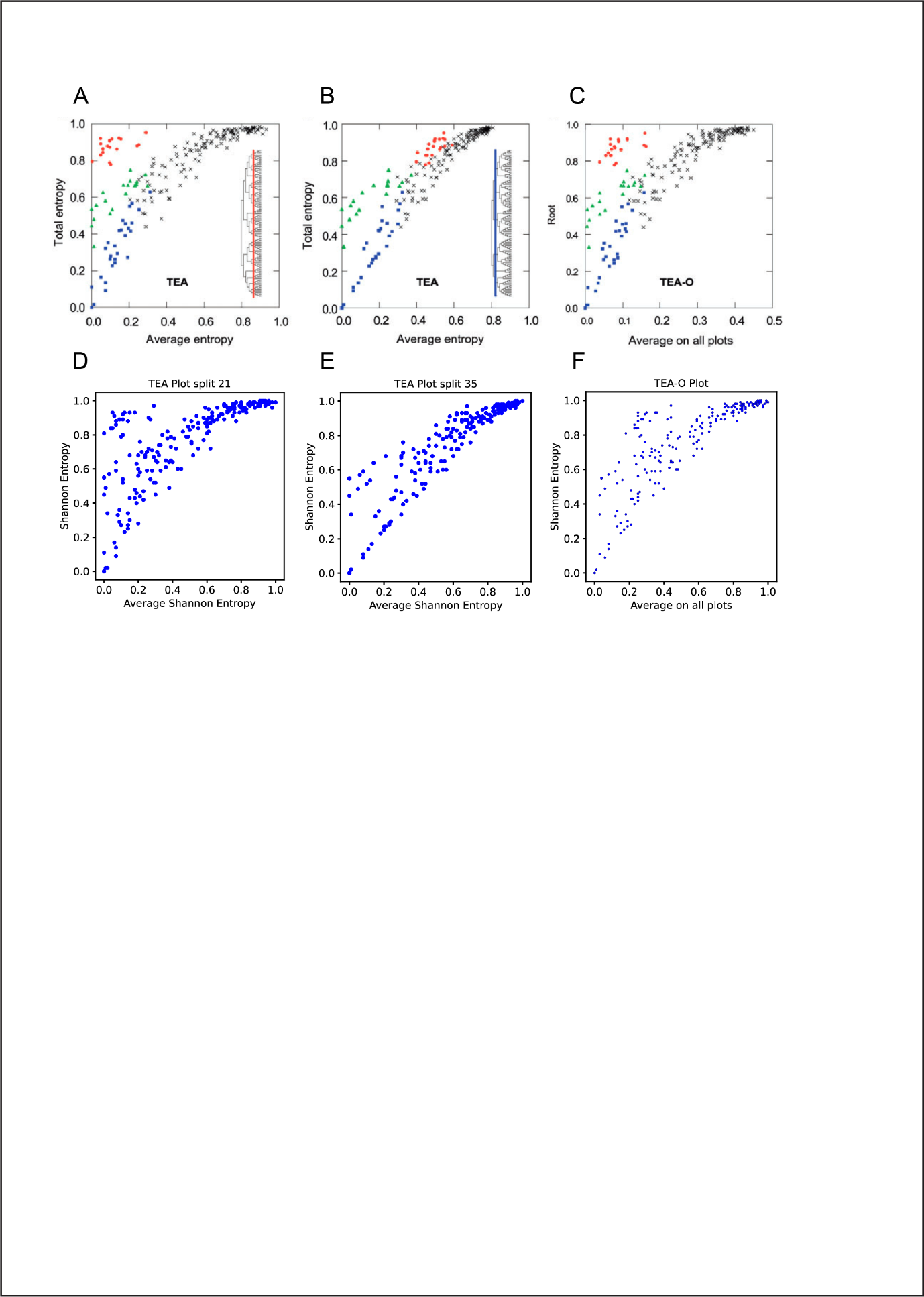


**Supplementary Figure 2**. Comparison of the original Two-Entropies Analysis with a synthetic dataset as defined by Ye et al. (Ye et al., 2008) and our re-implementation. **(A)** TEA mode (third generation) of the original analysis. **(B)** TEA mode (second generation) of the original analysis. **(C)** TEA-O mode original analysis. **(D)** TEA mode (third generation) our results. **(E)** TEA mode (second generation) our results. **(F)** TEA-O mode our results.

**Supplementary Table 3.** Summary of human OTUD7B (UniProt ID: Q6GQQ9) functional and structural features.

| **Position** | **Residue** | **Annotation** |
| --- | --- | --- |
| 191 | D | Active Site Residue |
| 194 | C | Active Site Residue |
| 197 | H | Site-Regulatory Residue |
| 358 | H | Active Site Residue |
| 802 | C | Binding Site Residue |
| 807 | C | Binding Site Residue |
| 819 | C | Binding Site Residue |
| 822 | C | Binding Site Residue |
| 50-88 |  | Disordered Region |
| 152-401 |  | TRAF-binding Domain |
| 167-440 |  | Catalytic Domain |
| 183-365 |  | OTU-Domain |
| 187-193 |  | Regulatory Loop |
| 442-587 |  | Disordered Region |
| 483-498 |  | Nuclear Localization |
| 652-711 |  | Disordered Region |
| 732-792 |  | Disordered Region |
| 796-831 |  | Zinc Finger |

**Supplementary Figure 3**. Output of PyTEA-O for OTUD7B case-study, based on a MSA of 5000 sequences.


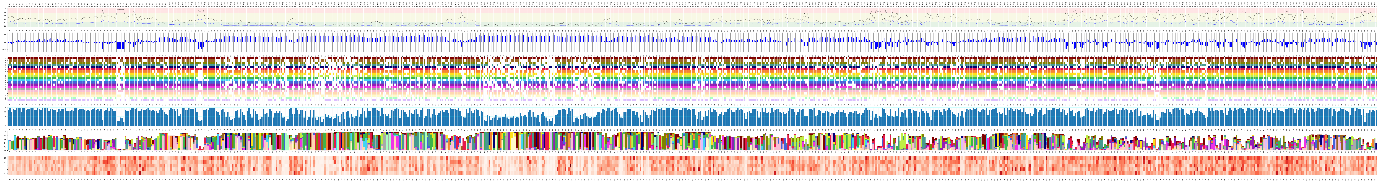


**Supplementary Table 4.** HDOCK modeling results for OTUD7B position 657 substitutions and controls. C194A was used as a positive control and E448Awas used as a negative control. Results include docking score, confidence score, and ligand RMSD (Å). ∆G and K_d_ estimates were obtained using PRODIGY.

| **Substitution** | **HDOCK docking score** | **HDOCK confidence score** | **Ligand RMSD (Å)** | ∆**G (kcal/mol)** | **K_d_ (M) at 25** *^◦^***C** |
| --- | --- | --- | --- | --- | --- |
| I (WT) | -235 | 0.85 | 37.2 | -7.9 | 1.6e-06 |
| A | -245 | 0.87 | 45.1 | -11.7 | 2.7e-09 |
| E | -217 | 0.79 | 48.4 | -10.2 | 3.2e-08 |
| G | -217 | 0.79 | 48.4 | -10.2 | 3.2e-08 |
| L | -259 | 0.90 | 24.3 | -8.6 | 4.9e-07 |
| M | -239 | 0.85 | 53.6 | -10.5 | 2.1e-08 |
| N | -272 | 0.92 | 60.7 | -9.2 | 1.7e-07 |
| P | -270 | 0.92 | 108.0 | -5.5 | 8.5e-05 |
| S | -217 | 0.79 | 48.4 | -10.2 | 3.2e-08 |
| T | -2167 | 0.79 | 48.4 | -10.2 | 3.2e-08 |
| V | -217 | 0.79 | 48.4 | -10.2 | 3.2e-08 |
| D191L (PC) | -234 | 0.84 | 41.7 | -6.6 | 1.5e-05 |
| E448A (NC) | -247 | 0.87 | 52.6 | -11.5 | 3.5e-09 |
